# ZrO_*x*_ Negative Capacitance Field-Effect Transistor with Sub-60 Subthreshold Swing Behavior

**DOI:** 10.1186/s11671-020-03468-w

**Published:** 2021-02-02

**Authors:** Siqing Zhang, Huan Liu, Jiuren Zhou, Yan Liu, Genquan Han, Yue Hao

**Affiliations:** State Key Discipline Laboratory of Wide Band Gap Semiconductor Technology, School of Microelectronics, Xidian University, Xi’an, 710071 China

**Keywords:** Amorphous ZrO_*x*_, Ferroelectric, FET, Subthreshold swing, Negative capacitance

## Abstract

Here we report the ZrO_*x*_-based negative capacitance (NC) FETs with 45.06 mV/decade subthreshold swing (SS) under ± 1 V *V*_GS_ range, which can achieve new opportunities in future voltage-scalable NCFET applications. The ferroelectric-like behavior of the Ge/ZrO_*x*_/TaN capacitors is proposed to be originated from the oxygen vacancy dipoles. The NC effect of the amorphous HfO_2_ and ZrO_*x*_ films devices can be proved by the sudden drop of gate leakage, the negative differential resistance (NDR) phenomenon, the enhancement of *I*DS and sub-60 subthreshold swing. 5 nm ZrO_*x*_-based NCFETs achieve a clockwise hysteresis of 0.24 V, lower than 60 mV/decade SS and an 12% *I*DS enhancement compared to the control device without ZrO_*x*_. The suppressed NC effect of Al_2_O_3_/HfO_2_ NCFET compared with ZrO_*x*_ NCFET is related to the partial switching of oxygen vacancy dipoles in the forward sweeping due to negative interfacial dipoles at the Al_2_O_3_/HfO_2_ interface.

## Background

As complementary metal oxide semiconductor (CMOS) devices scaling down constantly, the integrated circuit (IC) technique has entered into the era of “more than Moore era”. The driving force of IC industry and technology becomes the reduction of power consumption, instead of the miniaturization of transistors [1, 2]. However, the Boltzmann tyranny of MOSFETs, more than 60 mV/decade SS has restricted the energy/power efficiency [3]. In recent years, many proposed novel devices have the ability to achieve sub-60 mV/decade threshold swing, including impact ionization MOSFETs, tunnel FETs and NCFETs [4–7]. Due to the simple structure, the steep SS and improved drive current, NCFETs with a ferroelectric (FE) film have been regarded as an attractive alternative among these emerging devices [8–10]. The reported experiments on NCFETs mainly include PbZrTiO_3_ (PZT), P(VDF-TrFE) and HfZrO_*x*_ (HZO) [11–17]. However, the high process temperature and undesired gate leakage current along the grain boundaries of polycrystalline ferroelectric materials have restricted their development for the state-of-the-art technology nodes [18–26]. Recently, ferroelectricity in the amorphous Al_2_O_3_ and ZrO_*x*_ films enabled by the voltage-modulated oxygen vacancy dipoles has been investigated [27–29]. Compared with the crystalline counterpart, the amorphous ferroelectric-like films have significant advantages in reduced process temperature and leakage current. Thus, there are mass researches on FeFETs with amorphous gate insulator for the non-volatile memory and analog synapse applications [27, 30–34]. However, the systematical investigation on one-transistor ZrO_*x*_-based NCFET has not been carried out.

In this work, Ge NCFETs with 5 nm ZrO_*x*_ ferroelectric dielectric layer and 5 nm Al_2_O_3_/HfO_2_ ferroelectric dielectric layer have been proposed, respectively. We experimentally observed sub-60 mV/decade steep slope in ZrO_*x*_ (5 nm) NCFET, which can be attributed to the NC effect of ZrO_*x*_ ferroelectric layer. And we analyzed the polarization *P* as function of applied voltage *V* for the Ge/ZrO_*x*_/TaN capacitors. The ferroelectric-like behavior of the Ge/ZrO_*x*_/TaN capacitors is induced by the voltage-induced oxygen vacancy dipoles. Moreover, we attributed the improved *I*_DS_ and the sudden drop of *I*_G_ in the Al_2_O_3_/HfO_2_ NCFETs and ZrO_*x*_ NCFETs to the NC effect. We also observed the NDR phenomenon in the Al_2_O_3_/HfO_2_ NCFETs and ZrO_*x*_ NCFETs. In addition, we further analyzed the physical mechanism of interfacial dipoles-induced decreased NC effect in the Al_2_O_3_/HfO_2_ NCFET. The ZrO_*x*_ NCFETs with sub-60 mV/decade steep slope, improved drain voltage and low operating voltage will be suit for the design of NCFETs with low power consumption in the “more than Moore era”.

## Methods

**Fig. 1:**
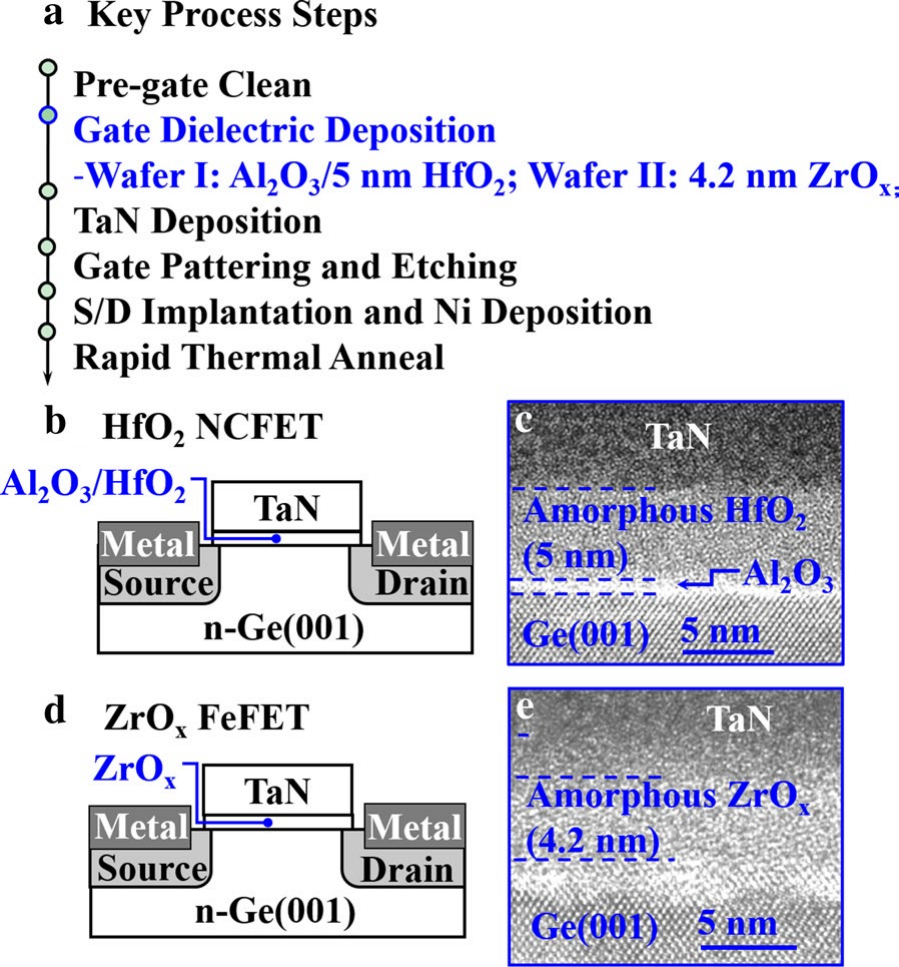
**a** Key process steps for the fabrication of the Al_2_O_3_/5 nm HfO_2_ NCFETs and 4.2 nm ZrO_*x*_ NCFETs. **b** Schematics and **c** HRTEM images of the fabricated ZrO_*x*_ NCFETs. **d** Schematics and **e** HRTEM images of the fabricated Al_2_O_3_/HfO_2_ NCFETs

Key process steps for NCFETs with ZrO_*x*_ and Al_2_O_3_/HfO_2_ fabrication are shown in Fig. 1a. Different gate dielectric insulators, including Al_2_O_3_/amorphous HfO_2_ (5 nm) films and amorphous ZrO_*x*_ (4.2 nm) films were grown on n-Ge (001) substrates by atomic layer deposition (ALD) at 300 °C. TMA, TDMAHf, TDMAZr and H_2_O vapor were used as the precursors of Al, Hf, Zr and O, respectively. The pulse time and purge time of the precursors of Hf and Zr are 1.6 s and 8 s, respectively. The pulse time and purge time of the precursors of Al are 0.2 s and 8 s, respectively. A TaN top gate electrode was then deposited on HfO_2_ or ZrO_*x*_ surfaces by reactive sputtering. Source/drain (S/D) regions were defined by lithography patterning and dry etching. After that, boron (B^+^) and nickel (Ni) was deposited in source/drain (S/D) regions. Finally, rapid thermal annealing (RTA) at 350 °C for 30 s in a 10^8^ Pa nitrogen ambient was carried out. Figure 1b, d show the schematics of the fabricated Al_2_O_3_/HfO_2_ NCFETs and ZrO_*x*_ NCFETs. High-resolution transmission electron microscope (HRTEM) image in Fig. 1c depicts the amorphous HfO_2_ (5 nm) film on Ge (001) with Al_2_O_3_ interfacial layer. HRTEM image in Fig. 1e depicts the amorphous ZrO_*x*_ (4.2 nm) film on Ge (001). The C–V curve of ZrO_x_ NCFETs and the X-ray photoelectron spectra (XPS) of TaN/ZrO_x_ (4.2 nm)/Ge capacitors were measured in Additional file 1: Fig. S1.

## Results and Discussion

**Fig. 2:**
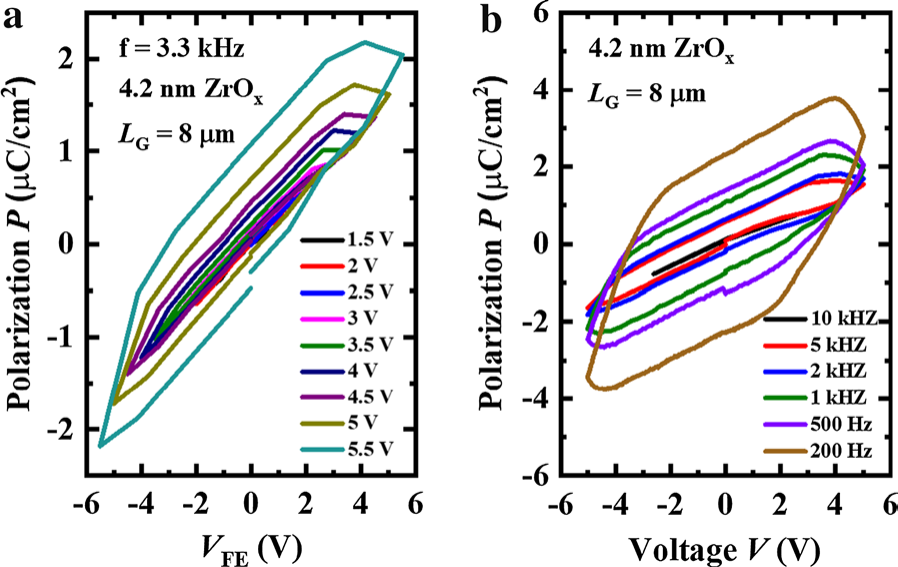
Measured *P* versus *V* characteristics of the 4.2 nm ZrO_*x*_ capacitors with **a** different sweeping ranges of *V* and **b** different measurement frequences

Figure 2a shows the measured curves of polarization *P v.s.* applied voltage *V* characteristics for the Ge/ZrO_*x*_/TaN capacitors at 3.3 kHz. The gate length (*L*_G_) of the capacitors are 8 μm. It is observed that the remnant polarization *P*_r_ of the Ge/ZrO_*x*_/TaN capacitors can be enhanced with larger sweeping range of *V*. The ferroelectric-like behavior of the amorphous ZrO_*x*_ film in the Fig. 2a is proposed to be originated from the voltage-driven oxygen vacancy dipoles [35]. Figure 2b shows the measured *P–V* curves for the Ge/ZrO_*x*_/TaN capacitors under different frequencies from 200 to 10 kHz. We can see that the ferroelectric-like behavior of the amorphous ZrO_*x*_ film remain stable for all frequencies. However, the *P*_r_ of the amorphous ZrO_*x*_ film is reduced with the increased frequencies. This phenomenon can be explained by the incomplete dipoles switching under high measurement frequencies [36, 37]. As measurement frequencies increasing, the time for the direction change of electric field in the amorphous ZrO_*x*_ film decreases. Thus, part of oxygen vacancy diploes switching is incomplete, providing decreased *P*_r_.

**Fig. 3:**
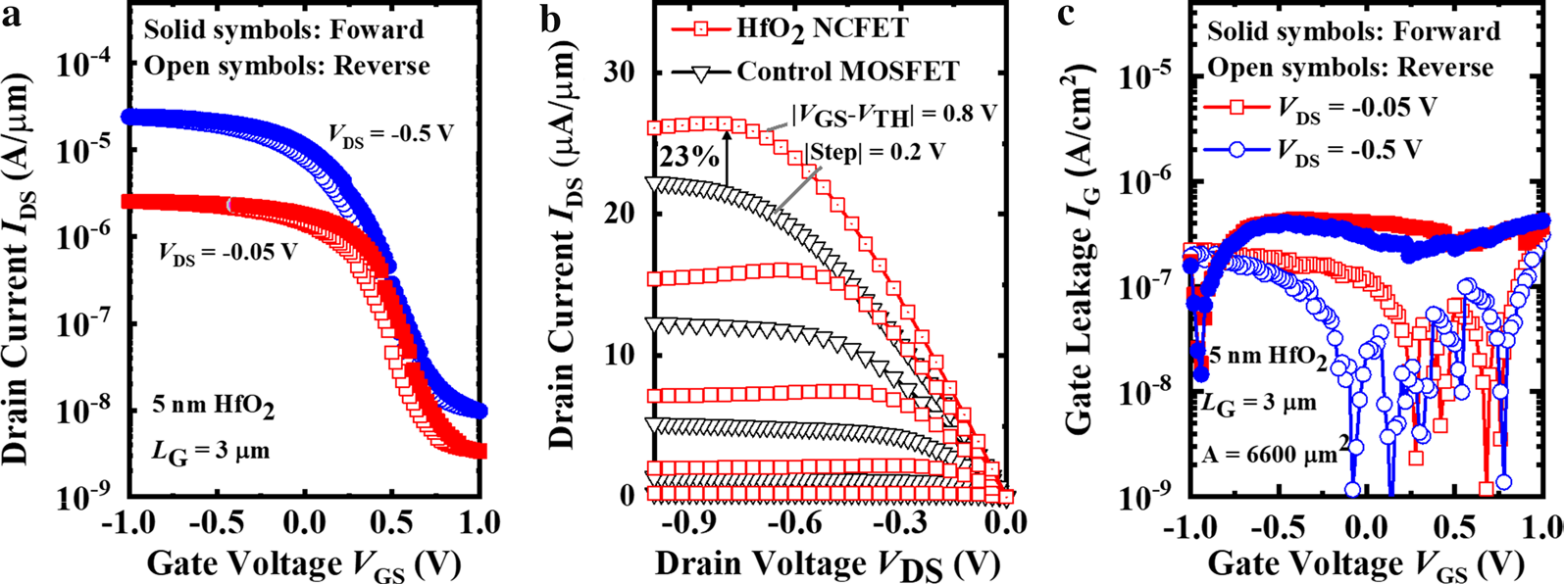
**a** Measured *I*_DS_–*V*_GS_ curves of the 5 nm HfO_2_ NCFET when *V*_DS_ = − 0.5 V and *V*_DS_ = − 0.05 V. **b** Measured *I*_DS_–*V*_DS_ curves of the HfO_2_ NCFET and the control MOSFET. **c** Measured *I*_G_–*V*_GS_ curves of the 5 nm HfO_2_ NCFET when *V*_DS_ = − 0.5 V and *V*_DS_ = − 0.05 V

Figure 3a shows the measured *I*_DS_–*V*_GS_ curves of a ferroelectric Al_2_O_3_/HfO_2_ NCFET at the *V*_DS_ of − 0.05 V and − 0.5 V. The *L*_G_ of the devices is 3 μm. The hysteresis loops of 0.14 V (*V*_DS_ = − 0.05 V, *I*_ds_ = 1 nA/μm) and 0.08 V (*V*_DS_ = − 0.5 V, *I*_ds_ = 1 nA/μm) are demonstrated, respectively. The clockwise hysteresis loops are attributed to the migration of oxygen vacancies and accompanied negative charges. The oxygen vacancy dipoles accumulate (deplete) in the Ge/Al_2_O_3_ interface under positive (negative) *V*_GS_. Therefore, the threshold voltage (*V*_TH_) increases (decreases) under forward (reverse) sweeping of gate voltages. As shown in Fig. 3b, the output characteristics of the Al_2_O_3_/HfO_2_ NCFET and the control FET are compared. The saturation current of the Al_2_O_3_/HfO_2_ NCFET exceeds 26 μA/μm, with a rise of 23% compared to that of the control FET at |*V*_GS_–*V*_TH_| =|*V*_DS_|= 0.8 V. The current enhancement is induced by the increased inversion charge intensity (*Q*_inv_) in the reverse polarization electric field and the amplification of surface potential [38, 39]. In addition to current enhancement, the obtained obvious NDR proves the NC effect of the amorphous HfO_2_ film. The NDR effect is caused by the incomplete switching of oxygen vacancy dipoles due to the coupling of drain-to-channel as *V*_DS_ increases [40, 41]. Figure 3c compares the measured gate leakage *I*_G_–*V*_GS_ curves for the 5 nm Al_2_O_3_/HfO_2_ NCFET at the *V*_DS_ of − 0.05 V and − 0.5 V. The sudden drops of *I*_G_ only during the reverse sweeping indicate the decreased voltage in the amorphous HfO_2_ film and the amplication of surface potential [42]. The absence of NC effect during the forward sweeping is caused by the partical switching of oxygen vacancy dipoles in the amorphous HfO_2_ film [43]. The different ability to contain oxygen atoms between Al_2_O_3_ and HfO_2_ layer leads to oxygen redistribution and negative interfacial dipoles at the Al_2_O_3_/HfO_2_ interface [44, 45]. Due to the presence of negative interfacial dipoles, it is difficult for the amorphous HfO_2_ film to realize complete polarization switching (NC effect) in the forward sweeping (Additional file 1).

**Fig. 4:**
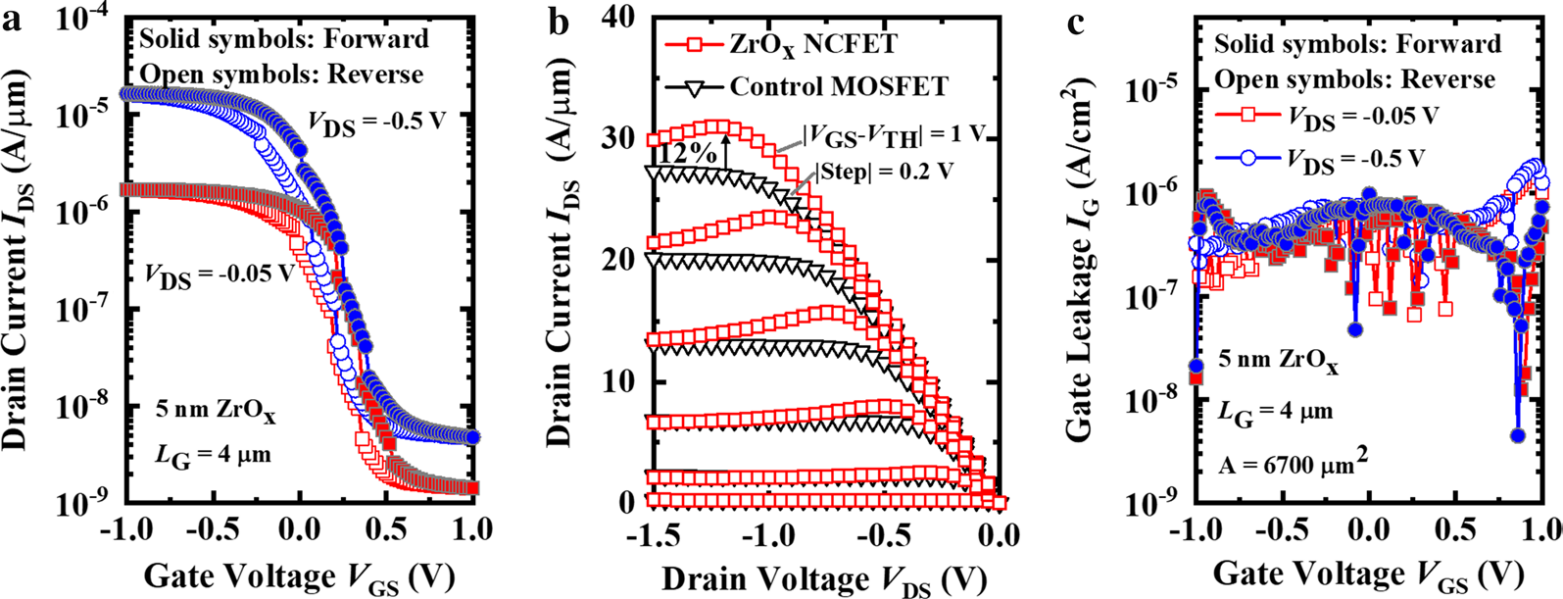
**a** Measured *I*_DS_–*V*_GS_ curves of the 5 nm ZrO_*x*_ NCFET when *V*_DS_ = − 0.5 V and *V*_DS_ = − 0.05 V. **b** Measured *I*_DS_–*V*_DS_ curves of the ZrO_*x*_ NCFET and the control MOSFET demonstraing the obvious NDR phenomenon. **c** Measured *I*_G_–*V*_GS_ curves of the 5 nm ZrO_*x*_ NCFET when *V*_DS_ = − 0.5 V and *V*_DS_ = − 0.05 V

Figure 4a shows the measured transfer curves of a ferroelectric ZrO_*x*_ NCFET at the *V*_DS_ of − 0.05 V and − 0.5 V. The *L*_G_ of the two devices are 4 μm. The clockwise hysteresis loops of 0.24 V (*V*_DS_ = − 0.05 V, *I*_ds_ = 1 nA/μm) and 0.14 V (*V*_DS_ = − 0.5 V, *I*_DS_ = 1 nA/μm) are demonstrated, respectively. As shown in Fig. 4b, the output characteristics of the ZrO_*x*_ NCFET and the control FET are compared. The saturation current of the ZrO_*x*_ NCFET exceeds 30 μA/μm, with a rise of 12% compared to that of the control FET at |*V*_GS_–*V*_TH_| =|*V*_DS_|= 1 V. The improved current enhancement and more obvious NDR indicate the enhanced NC effect of the amorphous ZrO_*x*_ film (5 nm) contrast to that of 5 nm HfO_2_ film. Figure 4c compares the measured gate leakage *I*_G_–*V*_GS_ curves for the 5 nm ZrO_*x*_ NCFET at the *V*_DS_ of − 0.05 V and − 0.5 V. Compared to the sudden *I*_G_ drops of Al_2_O_3_/HfO_2_ NCFET only during reverse sweeping in Fig. 3c, the sudden drops of *I*_G_ both in forward and reverse sweeping in Fig. 4c also prove the enhanced NC effect in the amorphous ZrO_*x*_ film.

**Fig. 5:**
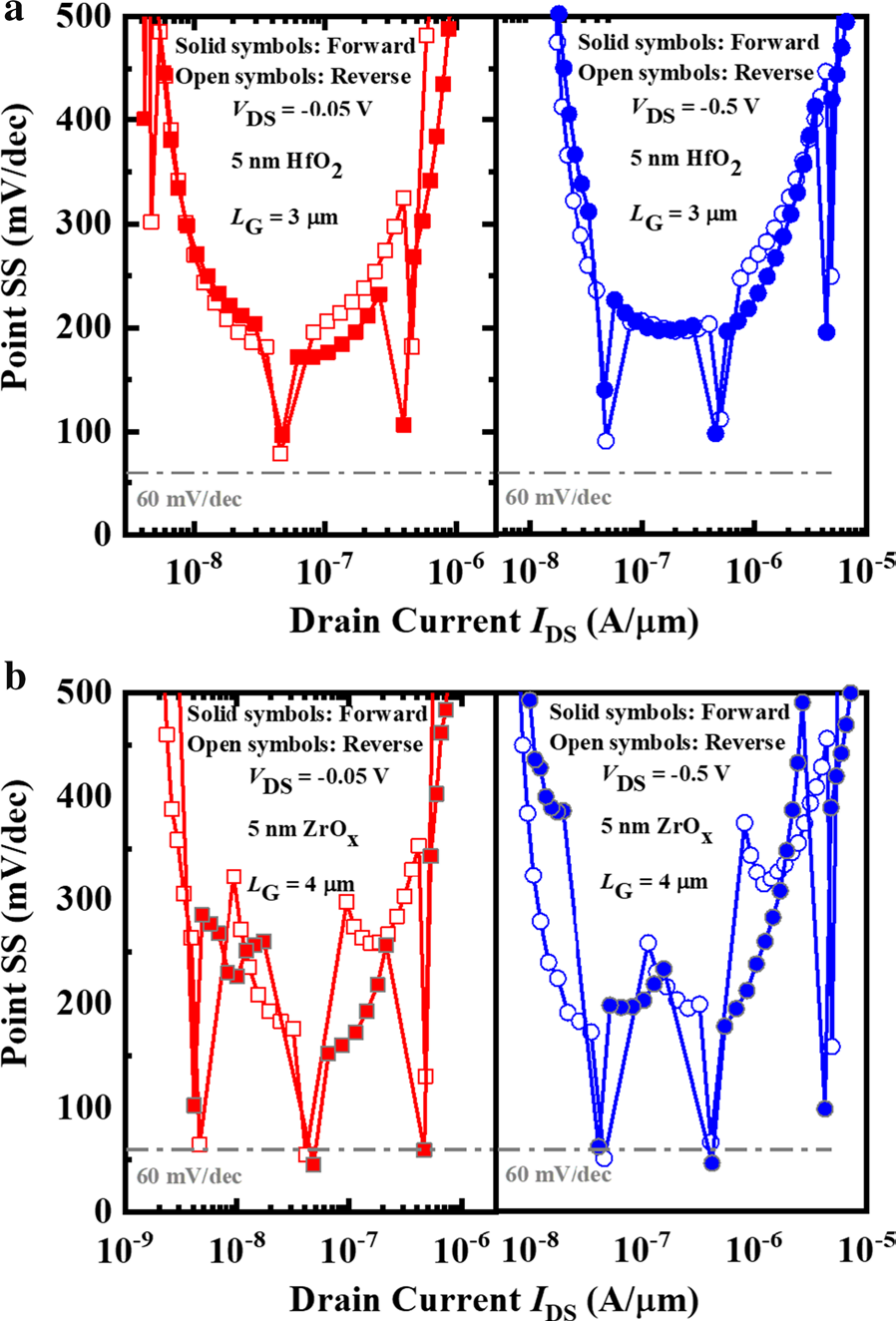
Point SS as a function of *I*_DS_ for the **a** Al_2_O_3_/5 nm HfO_2_ NCFETs and **b** 5 nm ZrO_*x*_ NCFETs

Figure 5a, b shows the point SS as function of *I*_DS_ for the Al_2_O_3_/HfO_2_ and ZrO_*x*_ NCFET at the *V*_DS_ of − 0.05 V and− 0.5 V. As shown in Fig. 5b, sub-60 mV/decade subthreshold swing (SS) can be achieved during forward or reverse sweeping of *V*_GS_ at the *V*_DS_ of− 0.05 V and− 0.5 V. When *V*_DS_ is− 0.05 V, a point forward SS of 45.1 mV/dec and a point reverse SS of 55.2 mV/dec were achieved. When *V*_DS_ is− 0.5 V, a point forward SS of 51.16 mV/dec and a point reverse SS of 46.52 mV/dec were achieved. Due to the different ability of scavenging effect for the Al_2_O_3_/HfO_2_ and ZrO_*x*_ layer, the partical dipoles switching is caused in the Al_2_O_3_/HfO_2_ NCFET. Therefore, the more obvious NC effect with sub-60 mV/decade SS is achieved in 5 nm ZrO_*x*_ NCFET.

## Conclusions

We report the demonstration of ferroelectric NC ZrO_*x*_ pFETs with the sub-60 mV/decade SS, low operating voltage of 1 V and a hysteresis of less than 60 mV. The impact of the amorphous ZrO_*x*_ films on the ferroelectric behavior is explained by the oxygen vacancy dipoles. The improved *I*_DS_ and NDR phenomenon are also obtained in Al_2_O_3_/HfO_2_ NCFETs and ZrO_*x*_ NCFETs compared to the control device. The suppressed NC effect of the Al_2_O_3_/HfO_2_ NCFET can be attributed to partical dipole swiching due to interfical dipoles at the Al_2_O_3_/HfO_2_ interface. The ZrO_*x*_ NCFETs with sub-60 mV/decade steep slope, improved drain voltage and low operating voltage pave a new way for future low power consumption NCFETs design.

## Supplementary Information

**Additional file 1**. From the C–V curve of ZrO_x_ NCFETs in Fig. S1 (a), we can see that the threshold voltage of the ZrO_x_ NCFETs is around 0.5 V. From the XPS of TaN/ZrO_x_ (4.2 nm)/Ge capacitors in Fig. S1 (b), we can see that a TaO_x_ interfacial layer formed in the TaN/ZrO_x_ interface and oxygen vacancies (ZrO_x_) in ZrO_x_ because of the scavenging effect.

## Data Availability

The datasets supporting the conclusions of this article are included in the article.

## Abbreviations

TaN: Tantalum nitride
ZrO_*x*_: Zirconium dioxide
TDMAZr: Tetrakis (dimethylamido) zirconium
*P*_r_: Remnant polarization
*E*_c_: Coercive electric field
MOSFETs: Metal–oxide–semiconductor field-effect transistors
Ge: Germanium
ALD: Atomic layer deposition
B^+^: Boron ion
Al_2_O_3_: Aluminum oxide
HRTEM: High-resolution transmission electron microscope
Ni: Nickel
RTA: Repaid thermal annealing
*I*_DS_: Drain current
*V*_GS_: Gate voltage
*V*_TH_: Threshold voltage
NCFET: Negative capacitance field-effect transistor
