# Supplementary material for: ZrOx Negative Capacitance Field-Effect Transistor with Sub-60 Subthreshold Swing Behavior
Source: Nanoscale Res Lett. 2021 Feb 2;16:21. doi: 10.1186/s11671-020-03468-w (PMC7855158; doi:10.1186/s11671-020-03468-w)
Supplement: Supplementary file 1 — Additional file 1. From the C–V curve of ZrOx NCFETs in Fig. S1 (a), we can see that the threshold voltage of the ZrOx NCFETs is around 0.5 V. From the XPS of TaN/ZrOx (4.2 nm)/Ge capacitors in Fig. S1 (b), we can see that a TaOx interfacial layer formed in the TaN/ZrOx interface and oxygen vacancies (ZrOx) in ZrOx because of the scavenging effect. [file 11671_2020_3468_MOESM1_ESM.docx]

**Supporting Information for:**

**ZrO_x_ Negative Capacitance Field-Effect Transistor with Sub-60 Subthreshold Swing Behavior**

Siqing Zhang^1, §^, Huan Liu^1, §^, Jiuren Zhou^1^, Yan Liu^1,^ *, Genquan Han^1^, and Yue Hao^1^

^1^ Wide Bandgap Semiconductor Technology Disciplines State Key Laboratory, School of Microelectronics, Xidian University, Xi’an 710071, China

* Corresponding authors.

E-mail addresses: xdliuyan@xidian.edu.cn


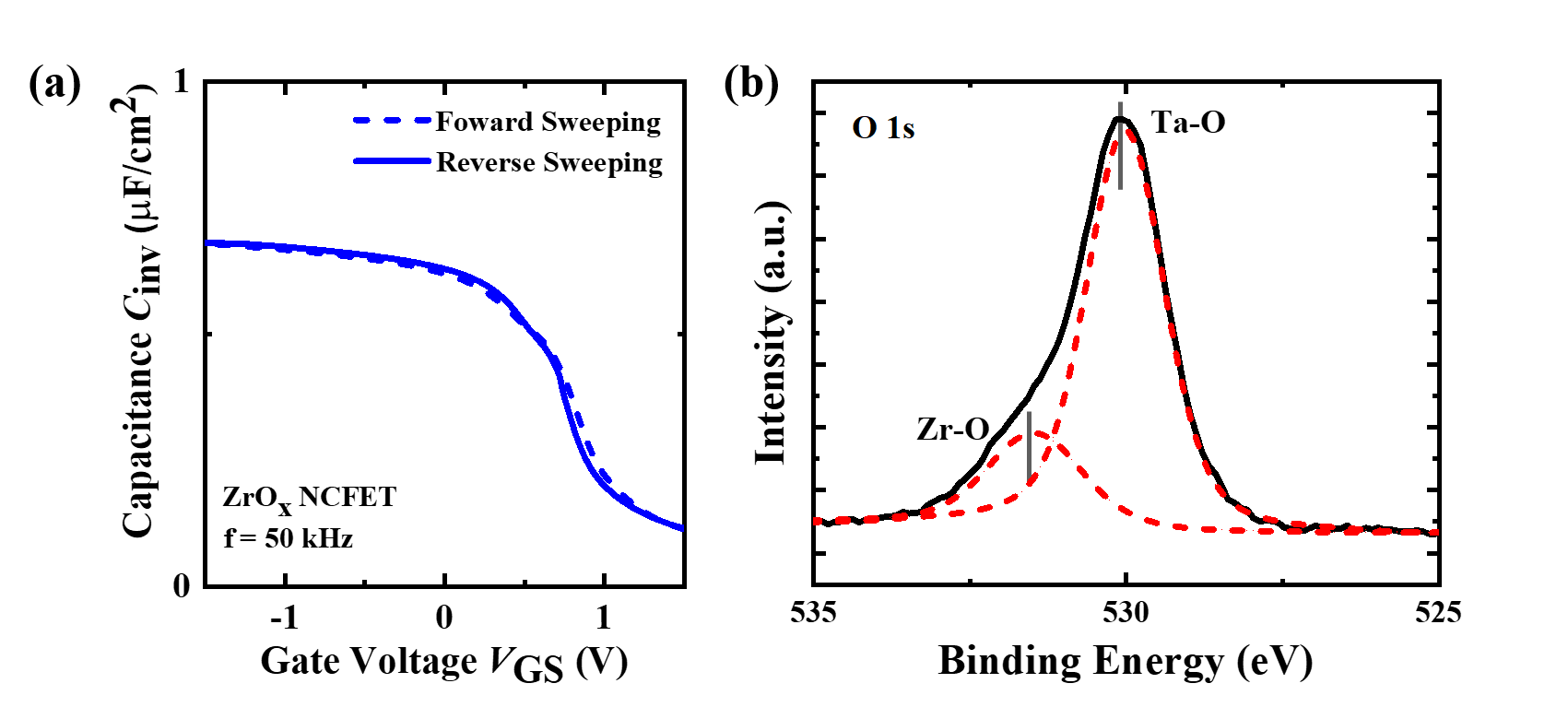


**Fig. S1** (a) the C-V curve of ZrO_x_ NCFETs. (b) the XPS of TaN/ZrO_2_ (4.2 nm)/Ge capacitors.
